# Antidepressant use and colorectal cancer risk: a Danish population-based case–control study

**DOI:** 10.1038/sj.bjc.6605911

**Published:** 2010-09-28

**Authors:** D P Cronin-Fenton, A H Riis, T L Lash, S O Dalton, S Friis, D Robertson, H T Sørensen

**Affiliations:** 1 Department of Clinical Epidemiology, Aarhus University Hospital, Olof Palmes Alle 43-45, 8200, Aarhus N, Denmark; 2 Department of Epidemiology, School of Public Health, Boston University, 715 Albany Street, TE3, Boston, MA, 02118, USA; 3 Institute of Cancer Epidemiology, Danish Cancer Society, Strandboulevarden 49, 2100, Copenhagen Ø, Denmark; 4 Department of Community and Family Medicine, Dartmouth-Hitchcock Medical Center, Lebanon, NH, USA

**Keywords:** colorectal cancer, selective serotonin reuptake inhibitors, tricyclic antidepressants, case–control study, pharmacoepidemiology

## Abstract

**Background:**

Earlier research suggests that use of selective serotonin reuptake inhibitors (SSRIs), but not tricyclic antidepressants (TCAs), reduces the risk of colorectal cancer (CRC).

**Methods:**

We conducted a population-based case–control study to investigate the association between antidepressant use and CRC risk. Cases were diagnosed with a first primary CRC from 1991 through 2008. We selected 10 population controls matched to cases on sex, birth year, and residence from the Danish Civil Registration System using risk-set sampling. We estimated the odds ratios (ORs) and 95% confidence intervals (CIs) associating antidepressant use with colorectal cancer occurrence, controlling for potential confounders.

**Results:**

The study included 9979 cases and 99 790 controls. We found no notable reduction in CRC risk in ever users (⩾2 prescriptions) of TCAs (OR=0.94; 95% CI: 0.84, 1.05), SSRIs (OR=0.97; 95% CI: 0.90, 1.05), or other antidepressants (OR=0.95; 95% CI: 0.83, 1.07). Associations for recent and former use of antidepressants were also near null. Intensity of antidepressant use (number of pills divided by total duration of use), regardless of duration, was not associated with CRC risk.

**Conclusions:**

We found no evidence that antidepressant use substantially reduces the risk of colorectal cancer.

Antidepressants are used to treat depression, anxiety, and pain (Alonso *et al*, 2004; Paulose-Ram *et al*, 2007). Most western countries report at least a doubling in the prevalence of antidepressant use over the last two decades (McManus *et al*, 2000; Paulose-Ram *et al*, 2007). Studies on the safety of antidepressants, including beneficial side effects, are important.

Antidepressants may have chemopreventive properties against colorectal cancer (CRC). They inhibit colorectal tumour growth in animal models and cell lines (Arimochi and Morita, 2006; Arimochi and Morita, 2008; Brandes *et al*, 1992; Steingart and Cotterchio, 1995; Tutton and Barkla, 1982). Few population-based studies, however, have focused specifically on CRC (Coogan *et al*, 2009; Haukka *et al*, 2010; Xu *et al*, 2006). A Canadian registry-based case–control study reported a reduced risk of CRC associated with daily selective serotonin reuptake inhibitor (SSRI) use for 0–5 years before CRC diagnosis or control's matched index date (incidence rate ratio (IRR)=0.70 (95% confidence interval (CI)=0.50, 0.96), but a near null association with tricyclical antidepressant (TCA) use (IRR=0.96 (95% CI=0.84, 1.10; Xu *et al*, 2006). A similar finding for SSRIs was reported in a US hospital-based case–control study (Coogan *et al*, 2009), but not in a Finnish registry-based cohort study (Haukka *et al*, 2010).

Given the high prevalence of antidepressant use and CRC in western countries (Parkin *et al*, 2005), any association between them would have significant public health implications. We therefore conducted a large population-based case–control study using prospectively collected prescription data to examine whether antidepressant use is associated with reduced risk of CRC.

## Patients and Methods

We conducted this population-based case–control study among the residents of Northern Denmark (the former counties of North Jutland and Aarhus), where approximately 20% (1.1 million inhabitants) of the Danish population lives. The Danish National Health Service provides tax-supported health care to all residents of the country and refunds part of patient expenditures for most prescribed drugs, including antidepressants.

All health-related services are linked to individual patients through their civil personal registration (CPR) number, assigned by the Danish Civil Registration System to all Danish residents since 1968. The CPR number encodes gender and date of birth and facilitates accurate linkage between Danish registries, including the Danish National Patient Registry (DNPR) and prescription databases (Gaist *et al*, 1997; Sørensen *et al*, 2009).

We used the DNPR to identify all patients aged at least 35 years whose first discharge diagnosis of CRC (ICD-8: 153.00–153.99, 154.00–154.19; ICD-10: C18.0–C18.9, C19.9, C20, and C20.9) occurred between 1 January 1991 and 31 December 2008 in North Jutland County or between 1 January 1998 and 31 December 2008 in Aarhus County. The DNPR contains detailed individual-level data on all non-psychiatric hospital admissions since 1977 and on all outpatient hospital contacts since 1995. Immediately after discharge from an inpatient hospitalisation or an outpatient clinic, the DNPR records the patient's CPR number, dates of admission and discharge, and up to 20 discharge diagnoses (Andersen *et al*, 1999). To ensure availability of at least 2 years of prescription data for each case, we included only CRC patients who resided in the study area for at least 2 years before their diagnosis date, yielding a total of 9979 CRC cases. The CRC diagnoses recorded in the DNPR before 1991 for North Jutland and before 1998 for Aarhus County were excluded from the study.

To identify controls, we accessed the Danish Civil Registration System, which maintains electronic records of all changes in vital status, migration, and residential address among Danish residents (Frank, 2000; Pedersen *et al*, 2006). We selected 10 unique population controls for each case, matched to cases on birth year, gender, and residence (county through 2006 and region after 2006; Sørensen *et al*, 2009), using risk-set sampling (Rothman *et al*, 2008). Risk-set sampling requires that controls be alive and at risk of a first hospital admission for CRC at the time the corresponding case was diagnosed. We assigned this date as the index date to both the case and all of the case's matched controls. We identified a total of 99 790 population controls, sampling only among individuals who were residents of the study area for at least 2 years before the index date.

We ascertained use of antidepressants from the prescription databases of two former Danish counties, North Jutland, and Aarhus (as noted above, local governments were organised into regions rather than counties in 2006). The North Jutland database was established in 1989 (with complete coverage since 1991), whereas that of Aarhus County was established in 1996 (with complete coverage since 1998; Sørensen *et al*, 2009). Thus for North Jutland, prescription data were available from 1 January 1989, whereas for Aarhus data were available from 1 January 1996. Matching on residence assures no bias from the differences in time periods with available prescription databases. Both databases include the CPR number, the type and quantity (including tablet and package sizes) of the prescribed drug according to the Anatomical Therapeutic Chemical (ATC) classification system (World Health Organisation Collaborating Centre for Drug Statistics Methodology, 2001) and the date the prescription was filled. We identified prescriptions for SSRIs, TCAs, and ‘other antidepressants.’ The following preparations were available during the study period: SSRIs: fluoxetine (N06AB03), citalopram (N06AB04), paroxetine (N06AB05), sertraline (N06AB06), fluvoxamine (N06AB08), escitalopram (N06AB10); TCAs (non-genotoxic; van Schaik and Graf, 1991; van Schaik and Graf, 1993): desipramine (N06AA01), imipramine (N06AA02), imipramine oxide (N06AA03), clomipramine (N06AA04), opipramol (N06AA05), trimipramine (N06AA06), doxepine (N06AA12), amoxapine (N06AA17), lofepramine (N06AA07); TCAs (genotoxic; van Schaik and Graf, 1991; van Schaik and Graf, 1993): amitriptyline (N06AA09), nortriptyline (N06AA10), protriptyline (N06AA11), dosulepine (N06AA16); and ‘other antidepressants’: maprotiline (N06AA21 and N06AC01), mianserine (N06AX03), duloxetine (N06AX), venlafaxine (N06AX16), mirtazapine (N06AX11), reboxetine (N06AX18), isocarboazide (N06AF01), and moclobemide (N06AG02).

The prescription databases also provided information on use of non-aspirin non-steroidal anti-inflammatory drugs (NSAIDs) and aspirin (ATC codes: M01A, N02BA01, N02BA51, and B01AC06; Cuzick *et al*, 2009; Ulrich *et al*, 2006), statins (ATC codes: C10AA01–C10AA07; Bonovas *et al*, 2007; Coogan *et al*, 2007; Singh *et al*, 2009), drugs used to treat diabetes mellitus as a marker of the disease (including insulin and oral hypoglycemics (ATC codes: A10B and A10A; Larsson *et al*, 2005; Renehan and Shalet, 2005), disulfiram as a proxy for chronic treated alcoholism (ATC code: N07BB01 ; Giovannucci *et al*, 1995; Glynn *et al*, 1996), neuroleptics (ATC code: N05A ; Dalton *et al*, 2006; Hippisley-Cox *et al*, 2007), and post-menopausal hormone replacement therapy (HRT ; ATC codes: G03C, G03D, G03F, and G03H B01; La Vecchia *et al*, 2005). These drugs were chosen because of their reported associations with CRC risk and potential associations with antidepressant use.

We obtained information from the DNPR on history of inflammatory bowel disease, which can be associated both with antidepressant treatment and with CRC (Mikocka-Walus *et al*, 2006); ulcerative colitis and Crohn's disease (ICD-8 codes: 563.01, 563.19, and 569.04; ICD-10 codes: K50.0, K50.1, K50.8, K50.9, or K51.0-K51.3). We also used the DNPR to identify previous hospital diagnoses for alcoholism as a second proxy for heavy alcohol use (ICD-8 code: 303; ICD-10 code: F10; Giovannucci *et al*, 1995; Glynn *et al*, 1996).

We excluded antidepressant drug use in the year before CRC diagnosis, to reduce the potential effect of undiagnosed disease on drug use. We measured drug intensity in terms of the median number of pills per day prescribed for each patient. We calculated all other exposures (ever/never use and temporality) on the basis of the total number of prescriptions filled during the exposure period. Accordingly, we defined ‘ever users’ as persons with at least two prescriptions during the entire exposure period and we defined ‘never/rare users’ as those with fewer than two prescriptions during the entire exposure period. To compare our drug exposure assessment with that used in earlier studies (Coogan *et al*, 2009; Haukka *et al*, 2010; Xu *et al*, 2006), we conducted a sensitivity analysis categorising ever users of antidepressants as persons with at least one antidepressant prescription.

We examined temporality of drug use by dividing ‘ever users’ into recent and former users. We defined recent users as those who had at least two prescriptions filled in the period 1 to 2 years before the index date and former users as those who had fewer than two prescriptions filled 1 to 2 years before the index date but at least two prescriptions filled during the entire observation period.

We also examined whether intensity of drug use (number of pills divided by the total duration of use) was associated with CRC risk. Duration was defined as the number of days from the date of a first prescription to the date of a last prescription plus the duration of the last prescription. We calculated the duration of the last prescription based on the prescription date and total number of pills dispensed. We divided duration of drug use into short term (1 year to <2 years), medium term (2 to 5 years) and long term (at least 5 years). We multiplied intensity of drug use by 100 to enable expression as a percentage. Categorisation of the intensity of drug use differed by antidepressant type. We grouped intensity of TCA use as low (<100%), medium (101–170%), and high (>171%); intensity of SSRI use as low (<70%), medium (71–100%), and high (>101%); and intensity of other antidepressant use as low (<100%) and high (>101%). We based the intensity groups on frequency of pills for each type of antidepressant. We further divided TCAs into genotoxic and non-genotoxic categories (van Schaik and Graf, 1991; van Schaik and Graf, 1993).

For potential confounding drugs, we categorised use as never/ rare, recent (1 to 2 years before diagnosis/index date), and former use (at least 2 years before the diagnosis/index date). The definition of never/rare use varied by medication. For NSAIDs, statins, neuroleptics, and hormone replacement therapy, never/rare use was considered less than two prescriptions throughout the observation period. For aspirin, less than 100 pills were considered never/rare use. We classified selective Cox-2 inhibitors and non-aspirin NSAIDs together (ATC codes: M01A^*^) in a group called ‘NSAIDs,’ and put aspirin (ATC codes: B01AC06, N02BA01, and N02BA51) into a separate group. For disulfiram, we considered only never/ever use.

### Statistical analyses

We calculated the frequency and proportion of cases and controls in categories of demographic variables, antidepressants use, potentially confounding drugs, and potentially confounding diseases. In all analyses, we used conditional logistic regression to compute odds ratios (OR) and associated 95% confidence intervals (95% CI) adjusted for the confounders discussed above. We used never or rare use (less than two prescriptions in total) as the reference group.

In our sensitivity analyses, ever users were defined as those with at least one antidepressant prescription. We carried out further analyses to investigate the association of continuous use of antidepressants with risk of CRC. We defined continuous use as individuals who were prescribed at least two antidepressants each year from the year of their first prescription to their last prescription. Finally, we conducted separate analyses for the risk of colon cancer and the risk of rectal cancer. Given the risk set sampling of controls, the ORs provide estimates of the corresponding incidence rate ratios in the underlying population. Analyses were performed using SAS version 9.13 (SAS Institute Inc., Cary, NC, USA).

## Results

Characteristics of the 9979 cases and 99 790 population controls are presented in Table 1. A slightly higher proportion of cases than controls had a history of diabetes and alcoholism. A lower proportion of cases than controls were recent users of NSAIDs, statins, or HRT, or were ever users of neuroleptics. Tricyclic antidepressants were used by 3.7% of cases and 4.0% of controls, SSRIs by 8.5% of cases and 8.9% of controls, and other antidepressants by 2.9% of cases and 3.1% of controls (Table 2). Median age of the study population was 72.4 years.

Ever use of TCAs, SSRIs, or other antidepressants was not notably associated with risk of CRC (OR=0.94 (95% CI: 0.84, 1.05); OR=0.97, (95% CI: 0.90, 1.05); and OR=0.95 (95% CI: 0.83, 1.07), respectively; Table 2). Associations for recent and former use were also near the null. Analyses combining duration and intensity of use also generated odds ratios near the null, regardless of antidepressant type (Table 3). As some individuals may have used drugs from all three classes, we repeated the analysis with a single model that included variables representing exposure to each drug group. There was little difference in the odds ratios generated by this model (data not presented). Individuals may also have switched from one antidepressant drug type to another. We therefore carried out additional analyses including individuals who used SSRI only, TCA only and both drug types in a model. There was little change in the odds ratios generated by this model (data not presented).

There was a slightly reduced risk of CRC associated with ever use of non-genotoxic TCAs (OR=0.88, 95% CI: 0.75, 1.04). Risk of CRC was not associated with ever use of genotoxic TCAs (OR=0.99, 95% CI: 0.83, 1.18) or use of both types of TCAs (OR=1.00, 95% CI: 0.75, 1.33). Risk estimates by type of TCA were all centred at the null for intensity, recency, and duration of use.

We found a slightly reduced risk of CRC associated with use of TCAs (OR=0.88, 95% CI: 0.73, 1.06) and SSRIs (OR=0.88, 95% CI: 0.75, 1.02) in our sensitivity analyses, with ever users defined as individuals with at least one antidepressant prescription.

We saw no evidence of an association between continuous use of antidepressants and CRC risk (data not presented).

## Discussion

Our findings show little evidence of a protective association between antidepressant use and CRC risk, regardless of antidepressant type. The odds ratios were near null for both recent and former use, and varied little by intensity and duration of use.

The validity of our estimates depends on several factors. We identified our study population from continuously updated population-based registries, with complete follow-up and high quality data. For example, cancer diagnoses in the DNPR have high sensitivity and specificity (Norgaard *et al*, 2005; Tetsche *et al*, 2005). Use of a population-based prescription registry, whose completeness approaches 100% (Gaist *et al*, 1997), ensured unbiased assessment of exposure before CRC diagnosis and eliminated recall bias. This design also facilitated adjustment for potential confounding drugs, diseases, and a more comprehensive list of potential confounders than examined in some earlier studies. The design also allowed assessment of a dose–response effect for antidepressant exposure in terms of intensity and duration of drug use.

Although our study was immune to recall bias by design, non-differential misclassification of antidepressant use was still an important limitation. Patients with depression are less likely to comply with prescription medications than those without depression (DiMatteo *et al*, 2000). However, our drug exposure assessment was based on redeemed prescriptions, for which patients had to collect the prescription and pay a portion of the drug cost. Our estimates are therefore likely to reflect actual use. The SSRIs were introduced to the Danish market about 1987 (Olfson *et al*, 2002). The prescription registries were established in 1989 and 1996, so we were unable to identify individuals who may have switched to SSRIs from TCAs before the study period. Although we controlled for many confounders, we were unable to adjust for body mass index, physical activity, or diet. We also had no information on the indication for antidepressant use or the severity of the underlying disease. Such confounding by indication (i.e., severity of depression) would underestimate the impact of antidepressant use on CRC risk. Of note, a large cohort study found an increased risk of CRC among women with the highest levels of depressive symptoms (Kroenke *et al*, 2005).

Our almost null results agree with the Finnish cohort study, which also found no reduction in CRC risk associated with SSRI use (Haukka *et al*, 2010). The Finnish study, however, did report a weak association between increased risk of CRC and >4 years of TCA use, which we did not observe. The Canadian also observed a slight increase in risk of CRC with long-term use of TCA (16–20 years; Coogan *et al*, 2009; Xu *et al*, 2006). We did not have such long-term data. In addition, our results are not so inconsistent with the Canadian and US studies, both of which reported a small reduction in the risk of CRC associated with SSRI use (Coogan *et al*, 2009; Xu *et al*, 2006), but their CIs were quite broad and included the null.

Our previous experience with the prescription data indicated quite a high prevalence of persons who fill just one antidepressant prescription, which suggests that these persons may not have completed taking even their first prescription. Therefore, to reduce misclassification from this non-compliance, we defined ever users as persons who had filled at least two prescriptions for antidepressants. In a sensitivity analyses, we defined ever use as redemption of one or more prescriptions instead of two or more prescriptions and observed a slight decrease in associations between CRC occurrence and antidepressant use more comparable with the results of the earlier case–control studies (Coogan *et al*, 2009; Xu *et al*, 2006). This sensitivity analysis suggests that the definition of ever use (one or more prescriptions) in the earlier studies contributed to the decreased risk of CRC associated with antidepressant use.

We found no evidence of an association between use of antidepressants and risk of colorectal cancer in this large population-based study.

## Figures and Tables

**Table 1:** Frequency distribution of demographic factors, comorbidities, and prescription medication use among colorectal cancer cases and matched controls in Northern Denmark, 1991–2008. (number, %)

	**Cases**		**Controls**	
	**N=9979**	**%**	**N=99 790**	**%**
*Gender*				
Women	4927	49	49 270	49
Men	5052	51	50 520	51
				
*Diabetes*				
No	9210	92	93 291	94
Yes	769	7.7	6499	6.5
				
*Alcoholism* a				
No	9719	97	97 587	98
Yes	260	2.6	2203	2.2
				
*Inflammatory bowel disease*				
No	9917	99	99 253	100
Yes	62	0.6	537	0.5
				
*NSAIDs*				
Never/rare use	6156	62	60 146	60
Recent use (1–3 years)	2330	23	25 187	25
Former use (3+ years)	1493	15	14 457	15
				
*Aspirin* b				
Never/rare use	7846	79	77 987	78
Recent use (1–3 years)	1892	19	19 374	19
Former use (3+ years)	241	2.4	2429	2.4
				
*Statins*				
Never/rare use	9268	93	92 061	92
Recent use (1–3 years)	688	6.9	7499	7.5
Former use (3+ years)	23	0.2	230	0.2
				
*Post-menopausal hormone replacement therapy*				
Never/rare use	8913	89	88 190	88
Recent use (1–3 years)	708	7.1	7673	7.7
Former use (3+ years)	358	3.6	3927	3.9
				
*Neuroleptics*				
Never/rare use	9424	94	93 686	94
Recent use (1–3 years)	343	3.4	4043	4.1
Former use (3+ years)	212	2.1	2061	2.1
				
*Marital status*				
Married	5649	57	56 602	57
Never married	698	7.0	7202	7.2
Divorced or widowed	3632	36	35 986	36

Abbreviation: NSAIDs=non-steroidal anti-inflammatory drugs.

a The definition of alcoholism included prescriptions of disulfiram as a proxy for chronic alcohol abuse.

b Low- and high-dose aspirin were grouped together.

**Table 2:** Ever/never use and temporality of antidepressant medication use among colorectal cancer cases and matched controls in Northern Denmark, 1991–2008 (number, %)

	***Cases***		**Controls**			
	**N=9979**	**%**	**N=99 790**	**%**	**OR** a	**95% CI**
*Tricyclic antidepressants*						
Never/rare use	9614	96	95 800	96	1.00	
Ever use	365	3.7	3990	4.0	0.94	0.84, 1.05
Temporality of use						
Never/rare use	9614	96	95 800	96	1.00	
Recent use (0 to <2 years)	184	1.8	2029	2.0	0.93	0.80, 1.09
Former use (2+ years)	181	1.8	1961	2.0	0.95	0.81, 1.11
						
*Selective serotonin reuptake inhibitors*						
Never/rare use	9128	92	90 900	91	1.00	
Ever use	851	8.5	8890	8.9	0.97	0.90, 1.05
						
Temporality of use						
Never/rare use	9128	92	90 900	91	1.00	
Recent use (1 to <2 years)	540	5.4	6693	6.7	0.97	0.88, 1.07
Former use (2+ years)	311	3.1	2197	2.2	0.97	0.86, 1.09
						
*Other antidepressants*						
Never/rare use	9689	97	96 654	97	1.00	
Ever use	290	2.9	3136	3.1	0.95	0.83, 1.07
						
Temporality of use						
Never/rare use	9689	97	96 654	91	1.00	
Recent use (1 to <2 years)	188	1.9	2007	2.0	0.96	0.83, 1.12
Former use (2+years)	102	1.0	1129	1.1	0.92	0.75, 1.13

Abbreviations: CI=confidence interval; NSAIDs=non-steroidal anti-inflammatory drugs; OR=odds ratio.

a Adjusted for diabetes, alcoholism, inflammatory bowel disease, use of NSAIDs, use of statins, use of hormone replacement therapy, use of aspirin, use of neuroleptics, and marital status.

**Table 3:** Duration and intensity of antidepressant medication use among colorectal cancer cases and matched controls in Northern Denmark, 1991–2008 (number, %)

	**Cases**		**Controls**			
	**N=9979**	**%**	**N=99 970**	**%**	**OR** a	**95% CI**
*Tricyclic antidepressants*						
Short-term use						
Never/rare use	9614	96	95 800	96	1.00	
Low intensity	78	0.8	925	0.9	0.86	0.68, 1.09
Medium intensity	76	0.8	969	1.0	0.80	0.63, 1.01
High intensity	74	0.7	750	0.8	1.01	0.80, 1.29
Long-term use						
Low intensity	51	0.5	524	0.5	1.00	0.75, 1.34
Medium intensity	33	0.3	349	0.4	0.98	0.69, 1.41
High intensity	53	0.5	473	0.5	1.17	0.88, 1.56
						
*Selective serotonin reuptake inhibitors*						
Short-term use						
Never/rare use	9128	91	90 900	91	1.00	
Low intensity	191	1.9	1865	1.9	1.04	0.89, 1.21
Medium intensity	216	2.2	2229	2.2	0.98	0.85, 1.13
High intensity	230	2.3	2599	2.6	0.90	0.78, 1.03
Long-term use						
Low intensity	99	1.0	1075	1.1	0.94	0.76, 1.16
Medium intensity	62	0.6	648	0.7	0.97	0.74, 1.26
High intensity	53	0.5	474	0.5	1.13	0.85, 1.51
						
*Other antidepressants*						
Short-term use						
Never/rare use	9689	97	96 654	97	1.00	
Low intensity	109	1.1	1268	1.3	0.87	0.72, 1.07
High intensity	126	1.3	1212	1.2	1.07	0.88, 1.29
Long-term use						
Low intensity	39	0.4	407	0.4	0.99	0.71, 1.38
High intensity	16	0.2	249	0.3	0.67	0.40, 1.11

Abbreviations: CI=confidence interval; NSAIDs=non-steroidal anti-inflammatory drugs; OR=odds ratio.

a Adjusted for diabetes, alcoholism, inflammatory bowel disease, use of NSAIDs, use of statins, use of hormone replacement therapy, use of low-dose aspirin and high-dose aspirin, use of neuroleptics, and marital status.
