# Antidepressant use and colorectal cancer risk: a Danish population-based case–control study

**DOI:** 10.1038/bjc.2011.176

**Published:** 2011-05-24

**Authors:** D P Cronin-Fenton, A H Riis, T L Lash, S O Dalton, S Friis, D Robertson, H T Sørensen

**Correction to:** *British Journal of Cancer* (2011) **104**, 188–192; doi:10.1038/sj.bjc.6605911

Upon publication in early 2011, the authors noticed a small error in the Methods section regarding the ATC codes for genotoxic and non-genotoxic TCA drugs, which may have caused some confusion for readers. The corrected text is presented below:

The list of ATC codes for TCA drugs should read:

‘*TCAs* (*genotoxic*) (*van Schaik and Graf, 1991, 1993*): desipramine (N06AA01), imipramine (N06AA02), imipramine oxide (N06AA03), clomipramine (N06AA04), opipramol (N06AA05), trimipramine (N06AA06), doxepine (N06AA12), amoxapine (N06AA17), lofepramine (N06AA07); *TCAs* (*non-genotoxic*) (*van Schaik and Graf, 1991, 1993*): amitriptyline (N06AA09), nortriptyline (N06AA10), protriptyline (N06AA11), dosulepine (N06AA16)’.

